# Daratumumab and Nanobody-Based Heavy Chain Antibodies Inhibit the ADPR Cyclase but not the NAD^+^ Hydrolase Activity of CD38-Expressing Multiple Myeloma Cells

**DOI:** 10.3390/cancers13010076

**Published:** 2020-12-30

**Authors:** Natalie Baum, Ralf Fliegert, Andreas Bauche, Julia Hambach, Stephan Menzel, Friedrich Haag, Peter Bannas, Friedrich Koch-Nolte

**Affiliations:** 1 Institute of Immunology, University Medical Center Hamburg-Eppendorf, 20246 Hamburg, Germany; n.baum@uke.de (N.B.); j.hambach@uke.de (J.H.); s.menzel@uke.de (S.M.); haag@uke.de (F.H.); 2 Department of Biochemistry and Molecular Cell Biology, University Medical Center Hamburg-Eppendorf, 20246 Hamburg, Germany; r.fliegert@uke.de (R.F.); a.bauche@uke.de (A.B.); 3 Department of Radiology and Nuclear Medicine, University Medical Center Hamburg-Eppendorf, 20246 Hamburg, Germany; p.bannas@uke.de

**Keywords:** multiple myeloma, CD38, ecto-enzyme, NAD^+^ hydrolase, antibodies, heavy-chain antibodies, nanobodies

## Abstract

**Simple Summary:**

Multiple myeloma is a hematological malignancy of antibody-producing plasma cells in the bone marrow. Nucleotides released from cells in the tumor microenvironment act as inflammatory danger signals. CD38 and other enzymes on the surface of cancer cells hydrolyze these nucleotides to immunosuppressive mediators, thereby hampering anti-tumor immune responses. Daratumumab and other CD38-specific antibodies mediate killing of tumor cells by natural killer cells, macrophages, and the complement system. Here, we investigated whether CD38-specific antibodies also inhibit the enzyme activity of CD38-expressing tumor cells, thereby providing a potential second mode of action. Our results showed that daratumumab and nanobody-based heavy chain antibodies inhibit the ADPR cyclase but not the NAD^+^ hydrolase activity of CD38. Thus, there remains a need for better CD38-inhibitory antibodies.

**Abstract:**

The nucleotides ATP and NAD^+^ are released from stressed cells as endogenous danger signals. Ecto-enzymes in the tumor microenvironment hydrolyze these inflammatory nucleotides to immunosuppressive adenosine, thereby, hampering anti-tumor immune responses. The NAD^+^ hydrolase CD38 is expressed at high levels on the cell surface of multiple myeloma (MM) cells. Daratumumab, a CD38-specific monoclonal antibody promotes cytotoxicity against MM cells. With long CDR3 loops, nanobodies and nanobody-based heavy chain antibodies (hcAbs) might bind to cavities on CD38 and thereby inhibit its enzyme activity more potently than conventional antibodies. The goal of our study was to establish assays for monitoring the enzymatic activities of CD38 on the cell surface of tumor cells and to assess the effects of CD38-specific antibodies on these activities. We monitored the enzymatic activity of CD38-expressing MM and other tumor cell lines, using fluorometric and HPLC assays. Our results showed that daratumumab and hcAb MU1067 inhibit the ADPR cyclase but not the NAD^+^ hydrolase activity of CD38-expressing MM cells. We conclude that neither clinically approved daratumumab nor recently developed nanobody-derived hcAbs provide a second mode of action against MM cells. Thus, there remains a quest for “double action” CD38-inhibitory antibodies.

## 1. Introduction

The tumor microenvironment promotes the growth of multiple myeloma cells in bone marrow niches and hampers anti-tumor immune responses [1,2]. The tumor microenvironment is characterized by hypoxia, acidic pH, high concentration of disulfide reducing agents, and enzymes that convert the endogenous danger signals ATP and NAD^+^ into immunosuppressive adenosine [3,4,5].

CD38 is the first enzyme within the purinergic signaling cascade that, together with CD203 and CD73, catabolizes extracellular NAD^+^ to adenosine [6,7], whereas CD39 and CD73 catabolize ATP to adenosine [5]. Hence, CD38 is one of the enzymes implicated in catabolism of NAD^+^ to generate immunosuppressive adenosine [3]. The main activity of CD38 is the hydrolysis of NAD^+^ to ADP-ribose (ADPR) and nicotinamide [8,9,10]. Minor activities include the water-independent conversion of NAD^+^ to cyclic ADP-ribose (cADPR), an intramolecular reaction that uses the high energy bond between nicotinamide and the “northern” ribose to generate an intramolecular bond, while releasing nicotinamide. CD38 can also hydrolyze this newly formed bond, converting cADPR to ADPR. In intracellular compartments, CD38 can also catalyze a base exchange reaction using either NAD^+^ or NADP, by replacing the nicotinamide moiety with nicotinic acid. All these reactions are mediated by the same active site encompassing E226 W125 and W189 of the CD38 molecule [11,12]. CD38 is structurally related to an invertebrate enzyme, Aplysia californica cyclase [13,14,15,16,17]. In contrast to CD38, the cyclase rather than the hydrolase is the main activity of the Aplysia enzyme. Both enzymes share five conserved intramolecular disulfide bridges, while CD38 contains an additional disulfide bridge.

CD38 has attracted attention as a target for therapeutic antibodies and nanobodies in MM because it is highly expressed on the cell surface of most multiple myeloma cells [18,19,20]. Daratumumab, a monoclonal antibody isolated by Genmab from a CD38-immunized human IgH transgenic mouse [21], showed clinical efficacy in MM patients [22,23,24,25,26,27]. Several other CD38-specific antibodies have entered clinical trials, including isatuximab, MOR202, and TAK-079 [28,29,30,31]. Moreover, CD38-specific nanobodies and nanobody-derived humanized heavy chain antibodies, as well as nanobody-based chimeric antigen receptors (CARs) showed promising results in preclinical experiments [32,33,34,35,36]. Therapeutic antibodies directed against specific antigens on the myeloma cell surface are thought to promote anti-tumor immune responses via Fc-mediated effector functions, i.e., complement dependent cytotoxicity (CDC), antibody dependent cellular cytotoxicity (ADCC), and antibody dependent cellular phagocytosis (ADCP) [21,37,38,39,40,41]. In addition, some antibodies can directly induce apoptosis, e.g., by crosslinking of the target antigen. Others might block binding of a growth factor or checkpoint receptor on the surface of immune cells. Recently, the concept was endorsed that inhibition of adenosine-producing enzymes could help turn the tumor microenvironment from “cold”, i.e., immunosuppressive, to “hot” i.e., pro-inflammatory [7,42,43,44,45,46]. Moreover, it was proposed that CD38-specific antibodies that inhibit the enzymatic activity of CD38 might provide a second mode of action [3,19,38,47,48,49].

A fluorometric enzyme assay is commonly used to assess the effects of antibodies on the enzymatic activity of CD38 [3,49,50,51]. This assay uses NGD^+^ (which carries a guanine nucleobase instead of adenine) instead of NAD^+^ as substrate. The increased fluorescence of the product cGDPR can be measured simply by fluorimetry [52]. The product cGDPR is more stable than cADPR, since CD38 does not hydrolyze cGDPR as efficiently as cADPR. However, an inhibitory effect measured in this assay does not necessarily imply a similar inhibitory effect on the NAD-hydrolase activity of CD38, i.e., the activity that feeds directly into the adenosine generation pathway. It is thus unclear, whether this assay is sufficient to validate the conclusion that isatuximab and daratumumab display a second mode of action, by blocking the generation of adenosine in the tumor microenvironment.

The goal of our study was to establish appropriate assays for monitoring the different enzymatic activities of CD38 on the cell surface of tumor cells, and to assess the effects of CD38-specific antibodies on these activities. To this end, we adapted the fluorometric cGDPR assay as well as a high-pressure liquid chromatography (HPLC) assay to monitor the enzyme activities of living tumor cells expressing CD38. Our results indicated that daratumumab and some nanobody-based heavy chain antibodies do effectively inhibit the ADPR cyclase activity of CD38 but not its NAD^+^ hydrolase activity.

## 2. Results

### 2.1. CD38-Expressing Tumor Cells Display More NAD-Hydrolase than ADPR Cyclase Activity

**Figure 1:**
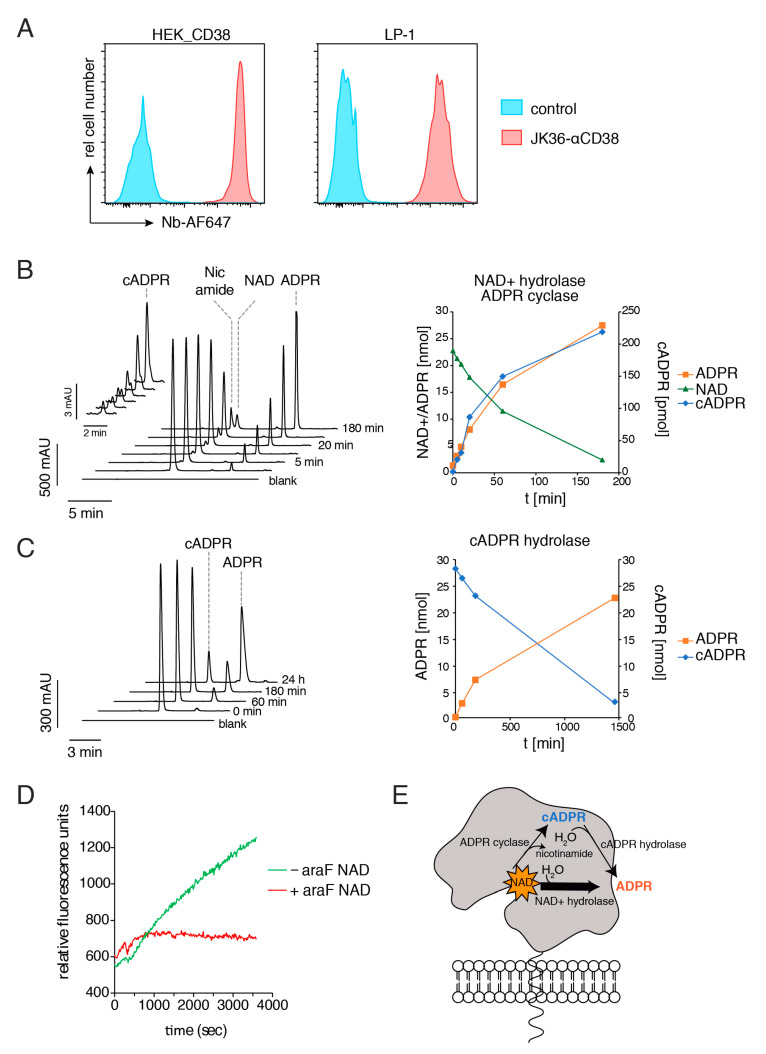
CD38-expressing tumor cells display potent NAD^+^ hydrolase activity and weak ADPR cyclase and cyclic-ADPR hydrolase activities. (**A**) HEK cells stably transfected with human CD38, untransfected HEK cells, and human LP-1 multiple myeloma cells were analyzed for cell surface levels of CD38, by incubation with Alexa647-conjugated CD38-specific nanobody JK36, before analysis by flow cytometry. (**B,C**) LP-1 cells were incubated at 37 °C in the presence of 500 μM NAD^+^ (b) or cADPR^+^ (c). The reaction was stopped at the indicated time points and NAD^+^ and its metabolites were quantified using reversed-phase HPLC. (**D**) LP-1 cells were incubated at 37 °C with 50 µM NGD^+^ and fluorescence intensity (ex/em: 300/410 nm) was determined using a microplate reader. (**E**) Schema summarizing the enzyme activities catalyzed by CD38.

To analyze the enzymatic reactions catalyzed by CD38-expressing tumor cells, we adapted an HPLC assay to measure the concentrations of NAD^+^, ADPR, and cADPR, after incubation of cells with NAD^+^ or cADPR (Figure 1). Cell surface levels of CD38 and of other nucleotide-metabolizing ecto-enzymes and receptors were measured on living cells by flow cytometry, using the fluorochrome-conjugated CD38-specific nanobody JK36 (Figure 1A). The results confirmed high cell surface levels of CD38 on both HEK cells stably transfected with CD38 and on LP-1 multiple myeloma cells endogenously expressing CD38, but only marginal levels of CD39, CD73, P2X7, or Adora2a. Consistently, neither LP-1 cells nor HEK_CD38 cells showed any detectable ATP-hydrolysis activity. Incubation of LP-1 cells with NAD^+^ resulted in a time-dependent decrease of extracellular NAD^+^, with a concomitant appearance of extracellular nicotinamide, ADPR, and cADPR (Figure 1B). Note that the levels of generated ADPR were approximately 100-fold higher than the levels of generated cADPR, consistent with the reported more potent NAD^+^ hydrolase activity of recombinant CD38, than its ADPR cyclase activity [12,14,53]. Pre-incubation of cells with the highly specific, irreversible, small molecule inhibitor of CD38, ara-F NAD^+^ [54], resulted in an almost complete inhibition of NAD^+^ hydrolase, ADPR cyclase, and cADPR-hydrolase activities, strongly suggesting that these activities are largely due to CD38.

Incubation of LP-1 cells with cADPR resulted in a time-dependent decrease of extracellular cADPR, with a concomitant appearance of extracellular ADPR (Figure 1C). The rate of cADPR hydrolysis was much slower than the rate of NAD^+^ hydrolysis (half of the input nucleotide turned over into ADPR within 12 h or 60 min, respectively), consistent with the reported more potent NAD^+^ hydrolase vs. cADPR hydrolase activity of CD38 [8,9].

We also assessed the GDPR cyclase activity of LP-1 cells using a fluorometric assay with NGD^+^ as substrate, instead of NAD^+^ (Figure 1D) [52]. The increased fluorescence of cGDPR vs. NGD^+^ can be monitored conveniently by fluorimetry, in a plate reader. The results showed a time-dependent increase of cGDPR. This was completely abrogated in the presence of the irreversible small molecule inhibitor of CD38, araF-NAD [54].

### 2.2. CD38-Specific Heavy Chain Antibodies Inhibit the GDPR Cyclase Activity of CD38-Expressing Tumor Cells More Potently than Daratumumab

**Figure 2:**
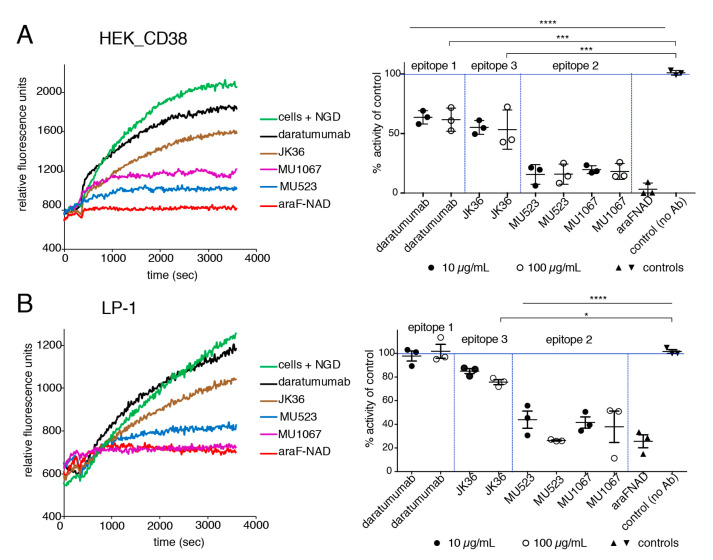
CD38-specific hcAbs MU523 and MU1067 potently inhibit the NGDR cyclase activity of human CD38-expressing tumor cells. The NGDR cyclase activity of CD38 expressing HEK cells (**A**) and LP-1 myeloma cells (**B**) was measured by the fluorimetric assay, as described in Figure 1D. Epitopes of daratumumab and hcAbs were assigned as described in [50]. Cells (1 × 10^5^ cells/well) were incubated with the indicated hcAbs (10 μg/mL or 100 μg/mL), daratumumab (20 μg/mL or 200 μg/mL), or araF-NAD (10 µM) for 20 min at 37 °C, before the measurement was started. After the first 20 cycles, 50 μM NGD^+^ was added and kinetic fluorescence reading (ex/em: 300/410 nm) was continued for 50 min. Cyclase activity was quantified by calculating the slope of curves of (A, B, left panel) during the linear phase, i.e., from *t* = 500 seconds to *t* = 1200 seconds (*n* = 3). Statistical analysis was performed using one-way ANOVA, followed by a Tukey post-hoc test for multiple comparisons. * *p* < 0.05; *** *p* < 0.001, **** *p* < 0.0001.

We previously selected CD38-specific nanobodies from immunized llamas that recognize three independent epitopes, designated epitope 1, 2, and 3 [50]. We observed that daratumumab blocks binding of epitope 1 nanobodies and vice versa, and that some epitope 2 nanobodies potently inhibited the GDPR cyclase activity of recombinant CD38 [50]. To analyze the effects of daratumumab and of nanobody-based hcAbs on the GDPR cyclase activity of living CD38-expressing tumor cells, we incubated CD38-transfected HEK cells or LP-1 cells with CD38-specific hcAbs, daratumumab, or araF-NAD, before addition of NGD^+^ (Figure 2).

The results showed a continuous increase of cGDPR during incubation of tumor cells with NGD^+^ in the absence of antibodies (green curves). Addition of araF-NAD effectively abrogates the increase of cGDPR, indicating that this is largely due to CD38 expression on the surface of the tumor cells (red curves). Addition of daratumumab slightly inhibited the GPDR cyclase activity of HEK cells but had little if any effect on the enzyme activity of LP-1 cells, whereas addition of epitope 2 hcAbs MU523 or MU1067 showed a potent inhibitory effect in both cell lines. Addition of the epitope 3 hcAb JK36 partially inhibited the increase of cGDPR in both cell lines. For better quantitative comparison, the slope of the curves during the linear phase, e.g., from *t* = 500 seconds to *t* = 1200 seconds was calculated at two different concentrations of antibodies (10 µg/mL, 100 µg/mL for hcAbs, 20 µg/mL, and 200 µg/mL for daratumumab) (Figure 2, right panels). The results showed that daratumumab, which binds epitope 1, did not have any detectable effect on the cyclase activity of LP-1 cells, even at a dose of 200 µg/mL. JK36-hcAb slightly reduced GDPR cyclase activity in both cell lines, whereas the epitope 2 hcAbs MU523 and MU1067 strongly inhibited GDPR cyclase activity in both cell lines.

### 2.3. CD38-Specific hcAb MU1067 Inhibits the CD38 Cyclase and cADPR Hydrolase Activities of the CD38 Expressing Tumor Cells but not their NAD^+^ Hydrolase Activity

In order to determine the effects of daratumumab and hcAbs on the enzyme activities of CD38-expressing tumor cells, we applied the HPLC assay described in Figure 1, to analyze the conversion of NAD^+^ to ADPR and cADPR, in the absence or presence of antibodies. In contrast to the NGDR cyclase assay described in the previous section, the HPLC assay is an end point assay and does not permit continuous monitoring of the substrate and reaction products. The reaction needs to be stopped by cooling the cells on ice, followed by a centrifugation step to separate the cells and the cell supernatants. Based on the kinetic analyses presented in Figure 1, we chose 60 min as the endpoint of analysis for NAD^+^, and 180 min as the endpoint of analysis for cADPR.

In order to assess whether the treatment of cells with the CD38-specific antibodies could induce internalization of CD38 and thereby contribute to the inhibition of enzyme activity, we incubated LP-1 cells for 3 h at 4 °C or at 37 °C with hcAb MU1067 or daratumab. Cells were then washed and assessed for cell surface levels of CD38, by staining with fluorochrom-conjugated hcAb JK36, an hcAb that binds to CD38 independent of both, MU1067 and daratumumab. The unabated high degree of cell surface staining with hcAb JK36 indicates that neither hcAb MU1067 nor daratumuab induce any significant internalization of CD38 during the time course of the experiment.

**Figure 3:**
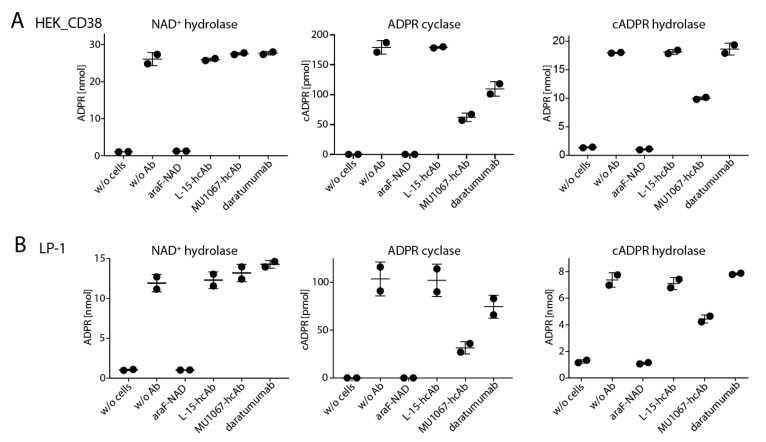
CD38-specific hcAb MU1067 and daratumumab inhibit the ADPR cyclase but not the NAD^+^ hydrolase activity of CD38-expressing LP-1 myeloma cells. HEK cells (**A**) or LP-1 cells (**B**) were pre-incubated for 15 min with the indicated antibodies or araF-NAD at 37 °C, before addition of NAD^+^ or cADPR, and further incubation for 60 min or 180 min at 37 °C, respectively. Nucleotide concentrations were measured by HPLC as in Figure 1. Toxin-specific L-15-hcAb was used as the control. Dots indicate values obtained in two independent experiments.

CD38-transfected HEK cells and LP-1 cells were preincubated with araF-NAD, hcAbs, or daratumumab for 15 min, before addition of NAD^+^ or cADPR, and further incubation to monitor NAD^+^ hydrolase and ADPR cyclase, or cADPR hydrolase activities, respectively. The cell supernatants were then analyzed by HPLC (Figure 3). The results showed that addition of araF-NAD completely prevented conversion of NAD^+^ to ADPR in both cell lines. However, neither daratumumab nor hcAbs showed any detectable inhibitory effect on the production of ADPR (A, B, first panel). Daratumumab, however, did slightly inhibit the production of cADPR (A, B, second panel) but not the hydrolysis of cADPR to ADPR (A, B, third panel). In contrast, hcAb MU1067 strongly inhibited both, the production and hydrolysis of cADPR, albeit not as effectively as araF-NAD (A, B, second and third panel, respectively).

### 2.4. The Reducing Agent Glutathione (GSH) Affects the Production of cADPR and ADPR by LP-1 Myeloma Cells in a Dose-Dependent Manner

**Figure 4:**
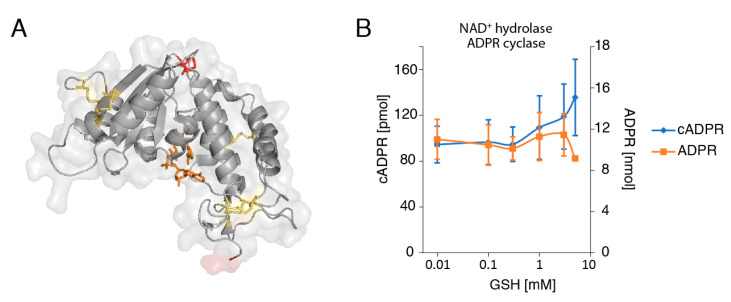
Glutathione enhances the production of cADPR by LP-1 myeloma cells in a dose-dependent manner. (**A**) Schematic illustrating the disulfide bonds in the extracellular domain of CD38. The five conserved disulfide bonds are depicted in yellow, the non-conserved disulfide bridge at C119/C201 is depicted in red. The N-terminal amino acid of the ectodomain of CD38 is shown in transparent dark red-five amino acids that separate this residue from the transmembrane domain of CD38. NAD^+^ in the active site crevice is depicted as an orange stick model. The model was made using the Pymol program and the coordinates of human CD38 in complex with NAD^+^ (pdb 2i65). (**B**) LP-1 cells were incubated for 10 min with the indicated concentrations of GSH before addition of NAD^+^ (500 μM) and further incubation for 60 min at 37 °C. Concentrations of ADPR and cADPR in cell supernatants were determined by HPLC, as in Figure 1.

CD38 has six disulfide bonds, five of which are conserved with the structurally-related ADPR cyclase of the Californian hermaphrodite sea slug, Aplysia californica (Figure 4). The extra non-conserved disulfide bond between C119 and C201 of CD38 (Figure 4A, shown in red) is located near the surface of the molecule on the opposite side of the NAD^+^-binding active site crevice. Akin to the redox-sensitive disulfide bonds on other membrane proteins, it is conceivable that this disulfide is sensitive to reduction in an inflammatory or tumor microenvironment. Glutathione (GSH) is a natural tripeptide (Glu-Cys-Gly), with a gamma peptide linkage between the carboxyl group of the glutamate side chain and cysteine. Cells maintain a high concentration of GSH (0.5–10 mM) in the cytosol and organelles, which can be released from cells during inflammation or tissue damage. We therefore used our HPLC assay to determine whether addition of glutathione could influence the enzymatic activities of CD38 expressing LP-1 cells. We incubated LP-1 with GSH for 10 min at 37 °C, followed by addition of NAD^+^ and further incubation for 60 min (Figure 4B). At concentrations below 1 mM, GSH had little if any effect on NAD^+^ catalysis by LP-1 cells. Treatment of cells with GSH at concentrations of 1–5 mM, however, resulted in an elevated production of cADPR, with a concomitant reduction in ADPR (Figure 4B). These effects were confirmed in three independent experiments, albeit with a high variability in the degree of elevated cADPR, as reflected by the large error bar in Figure 4B.

### 2.5. The Denaturing Reducing Agent DTT Inhibits the Conversion of NAD^+^ to ADPR and cADPR by CD38-Expressing Tumor Cells in a Dose-Dependent Manner

**Figure 5:**
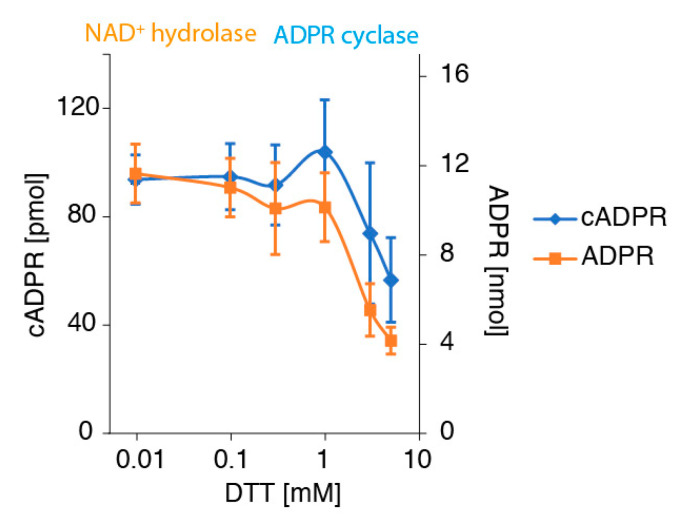
DTT inhibits the production of ADPR and cADPR by LP-1 cells in a dose-dependent manner. LP-1 cells were incubated for 10 min with the indicated concentrations of DTT before addition of NAD^+^ (500 μM) and further incubation for 60 min at 37 °C. Concentrations of ADPR and cADPR in cell supernatants were determined by HPLC, as in Figure 1.

Dithiothreitol (DTT) is a potent reducing agent commonly used in biochemistry to reduce disulfide bonds in proteins, e.g., during denaturing gel electrophoresis. DTT is also used to denature and inactivate CD38 on erythrocytes [55]. Treatment of CD38-expressing tumor cells with DTT, indeed, resulted in a dose-dependent inhibition of the conversion of NAD^+^ to both, ADPR and cADPR (Figure 5). At an intermediate concentration of 1 mM, treatment of cells with DTT resulted in a slightly elevated production of cADPR, with a concomitant reduction in ADPR, reminiscent of the effects of high concentrations of GSH (Figure 4B).

### 2.6. Daratumumab and CD38-Specific hcAb MU1067 Protect CD38 against the Denaturing Effects of DTT

**Figure 6:**
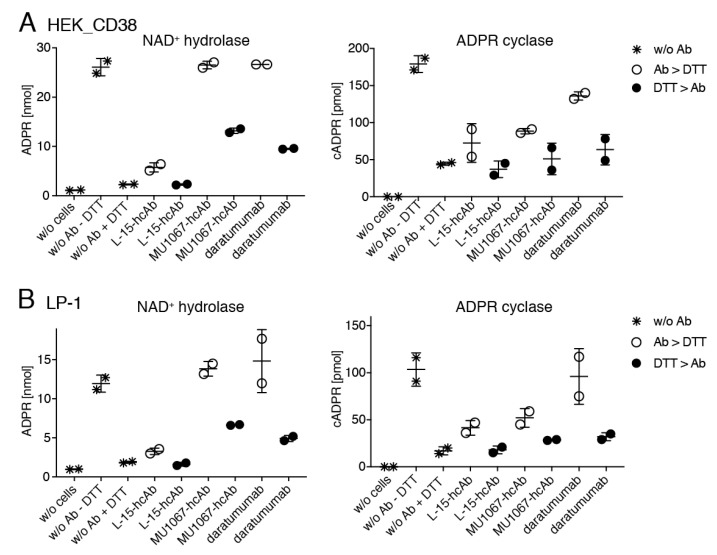
Daratumumab and hcAb MU1067 protect the NAD^+^ hydrolase and ADPR cyclase activities of the CD38-expressing tumor cells from inactivation by DTT. CD38-transfected HEK cells (**A**) and LP-1 cells (**B**) were pre-incubated with 5 mM DTT for 20 min before addition of antibodies and further incubation for 15 min, or vice versa. NAD^+^ was then added and incubation continued for 60 min at 37 °C. Concentrations of ADPR and cADPR were determined by HPLC, as in Figure 1. Toxin-specific L-15-hcAb was used as control.

In order to determine whether antibodies bound to CD38 could influence DTT-mediated denaturation of CD38, we incubated CD38-expressing tumor cells with 5 mM DTT and NAD^+^, in the presence or absence of daratumumab or hcAb MU1067 and analyzed the reaction products by HPLC (Figure 6). The results showed that pretreatment of cells with either daratumumab or hcAb MU1067 effectively protected CD38 from the detrimental effects of DTT. A protective effect was still discernible, albeit much weaker, when the antibodies were added 20 min after DTT.

## 3. Discussion

Antibodies that inhibit the enzyme activity of CD38 could help modulate the microenvironment of MM by preventing the conversion of pro-inflammatory NAD^+^ to immunosuppressive adenosine [3,37,50,51]. To better assess the inhibitory potency of CD38-specific antibodies, here we report the successful adaptation of HPLC and fluorimetric assays to monitor the enzyme activity of living CD38-expressing tumor cells, i.e., CD38-transfected HEK cells and the LP-1 multiple myeloma cell line.

The fluorimetric assay using NGD^+^ as a substrate was used in previous studies with recombinant CD38, because it allows the convenient use of a plate reader to monitor enzyme activity over time [37,50,51]. A drawback of this assay was that it provides information only about the GDPR cyclase but not the NGD^+^ hydrolase activity of CD38. By inference, this assay provides information only about the ADPR cyclase activity of CD38, but not its major NAD^+^ hydrolase activity. The HPLC assay has the drawback that it is more cumbersome to set up and that it allows only end-point measurements. The advantage of the HPLC assay is that it provides information about both, the major NAD^+^ hydrolase and the minor ADPR cyclase activity of CD38 [8,9,10,53]. Furthermore, the HPLC assay can also be used to assess the other minor activity of CD38, i.e., the hydrolysis of cADPR. The results of previous studies with the fluorimetric assay suggested that daratumumab is a weak antagonist and that epitope 2 nanobodies are strong antagonists of the cyclase activity of CD38 [50,51]. The results of our HPLC assay presented here support this conclusion. However, our results also revealed that neither daratumumab nor hcAb MU1067 inhibit the hydrolysis of NAD^+^ to ADPR by CD38, i.e., its major enzyme activity.

**Figure 7:**
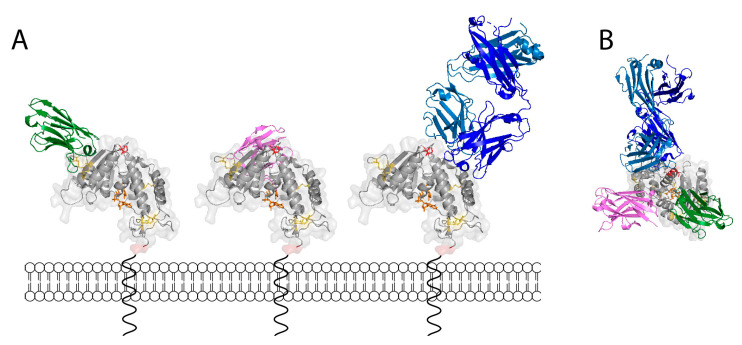
3D models of CD38 with bound nanobodies MU375, MU523, and a Fab fragment of isatuximab. 3D-models of CD38 in complex with the epitope 1 nanobody MU375 (green), epitope 2 nanobody MU523 (magenta), and the Fab fragment of isatuximab (heavy chain dark blue, light chain lighter blue) were generated by PyMol, using data from PDB files 5f21, 5f1o, and 4cmh. CD38 is depicted in the same orientation and color coding as in Figure 4 (with 5 conserved disulfide bonds in yellow, the non-conserved disulfide bond at C119/C201 in red, NAD^+^ in the active site crevice in orange, and the N-terminal amino acid of the extracellular domain in transparent red). The N-terminal cytosolic domain and transmembrane domain are depicted schematically. The models in (**A**) show CD38 as viewed in the plane of the cell membrane, the model in (**B**) shows CD38 as viewed from the “top” with the cell membrane “below” CD38.

Nanobodies reportedly display a propensity to bind to the active site crevice of enzyme antigens [56,57]. This was attributed to the longer CDR3 loop of nanobodies compared to the V-domains of conventional antibodies. Co-crystal structures of nanobodies with different enzymes revealed that the nanobody CDR3 loop can form a finger-like extension that reaches into and blocks the active site crevice [58]. However, available co-crystal structures of CD38 in complex with isatuximab or nanobodies (pdb codes 5f21, 5f1o, and 4cmh) show distinct binding sites at substantial distances away from the NAD-binding active site crevice (Figure 7). A co-crystal structure of daratumumab is not yet available, but peptide mapping and our own crossblockade analyses indicate that daratumumab also binds an epitope far from the active site crevice (overlapping with epitope 1 nanobodies). The co-crystal structure of CD38 in complex with the Fab fragment of isatuximab also indicates a binding site far from the active site crevice [37]. Isatuximab was shown to potently inhibit the GDPR cyclase activity of CD38. Unfortunately, isatuximab was not available for the current study, so that its effect on the NAD^+^ hydrolase activity of CD38 remains to be determined. The co-crystal structure of CD38 in complex with the inhibitory epitope 2 nanobody 523 [50] also suggests a binding site far from the active site crevice. Taken together, these results indicate that the inhibitory effect of these antibodies against the ADPR cyclase activity is through an allosteric mechanism, e.g., by stabilizing the structure of CD38, so as to prevent conformational changes that are required for its ADPR cyclase but not its NAD^+^ hydrolase activity.

CD38 contains an extra pair of cysteine residues compared to the Aplysia ADPR cyclase [14]. These cysteines form a disulfide bond on the surface of the molecule. It is tempting to speculate that this disulfide bond is sensitive to changes in the reducing conditions of the tumor microenvironment. We observed that treatment of MM cells with the reducing agent GSH stimulates the ADPR cyclase activity and concomitantly inhibits the NAD^+^ hydrolase activity of CD38-expressing LP-1 cells. Considering that the intracellullar levels of GSH lie in the range of 0.5–10 mM, millimolar concentrations of extracellular GSH might be expected only in the immediate vicinity of the lysed cells. Further studies should address the question whether the reducing conditions and other physicochemical features of the tumor microenvironment (pH, hypoxia) similarly effect the enzyme activities of CD38.

Future studies are required to determine whether daratumumab, nanobody-based heavy chain antibodies, or other CD38-specific therapeutic antibodies affect the enzymatic activities of CD38 in clinical settings. Importantly, our results showed that daratumumab and epitope 2 hcAbs stabilize CD38 against the detrimental effects of the biochemical reducing agent DTT. These results raise the possibility that daratumumab and other CD38-specific Abs might actually promote an immunosuppressive tumor microenvironment, i.e., by promoting CD38-mediated hydrolysis of NAD^+^, a crucial step in the pathway that generates adenosine from NAD^+^ [3,43].

In summary, we showed that daratumumab and hcAb MU1067 inhibit the ADPR cyclase but not the NAD^+^ hydrolase activity of CD38-expressing MM cells. Our results underscore the need for careful assessment of the different effects of therapeutic antibodies on the enzyme activities of CD38 on living MM cells. We conclude that neither clinically approved daratumumab nor recently developed nanobody-derived hcAbs provide a second mode of action against MM cells and that therefore there remains a quest for double action CD38-inhibitory antibodies. The assays reported here provide a basis for such studies.

## 4. Materials and Methods

Cell lines and antibodies: The human multiple myeloma cell line LP-1 was obtained from DSMZ (Braunschweig, Germany). HEK cells stably transfected with human CD38 were generated in our lab. Recombinant human CD38-specific heavy chain antibodies and an irrelevant toxin-specific heavy chain antibody were generated in our lab, as described previously [50,59]; araF-NAD (ara-2′-F-NAD^+^) was obtained from BioLog (Bremen, Germany), and GSH and DTT was obtained from Sigma (Frankfurt, Germany). The following antibodies were used to assess the expression of CD38 (HIT2, Biolegend, San Diego, CA, USA), CD39 (eBioA1, eBioscience, San Diego, CA, USA), CD73 (AD2, Becton Dickinson, Franklin Lakes, NJ, USA), CD203a (ab240832, Cambridge, UK), P2X7 (Nb 1C113, patent WO2013178783), and Adora2a (7F6-G5-A2, Novus Biologicals, Littleton, CO, USA).

CD38 internalization assay: LP-1 cells were incubated for 3 h at 4 °C or at 37 °C in the presence of hcAb MU1067 or daratumumab (10 µg/mL). Cells were washed and incubated for 15 min at 4 °C, with the Alexa647-conjugated hcAb JK36 [50], before analysis by flow cytometry (FACS Calibur, Becton Dickinson, Franklin Lakes, NJ, USA).

Fluorometric enzyme assays: CD38-transfected HEK or LP-1 cells (1 × 10^5^ cells/well) were incubated at 37 °C in the dark for 20 min, with daratumumab (20 or 200 µg/mL), hcAbs (10 or 100 µg/mL), or araF-NAD (10 µM), before fluorescence measurements. After recording for 20 cycles, NGD^+^ (50 µM, Sigma, St Louis, MO, USA) was added, followed by further incubation in the dark at 37 °C. Production of cGDPR was monitored continuously for 50 min at 410 nm (emission wavelength), with the excitation wavelength set to 300 nm, using a Tecan Infinite M 200 microplate fluorimeter. Readings (EX300/EM410) from wells without cells were subtracted from all sample readings and the values were plotted as the Relative Fluorescence Units (RFU) vs. time. As an estimate for the rate of cGDPR production, the slope of the curves (RFU/s) during the linear phase of the reaction, i.e., *t* = 500–1200 s, was calculated.

Luminometric assay for ATP-hydrolysis: CD38-transfected HEK or LP-1 cells (2 × 10^4^ cells/well) were incubated at 37 °C in the dark for 30 min with ATP (50 µM, Sigma, St Louis, MO, USA). The cells were removed by centrifugation and the supernatants were mixed with an equal volume of celltiter glo 2.0 reagent (Promega, Madison, WI, USA), incubated for 10 min luminometry, on a Victor microplate reader. CD39-transfected HEK cells were used as the positive control.

HPLC enzyme assays: CD38-transfected HEK-cells or LP-1 cells (1 × 10^5^ cells/well) were pre-incubated with antibodies, araF-NAD, GSH, or DTT, at the indicated concentrations and at times before addition of NAD^+^ (500 μM) or cADPR (500 μM) for 60 and 180 min, respectively, and further incubation at 37 °C with mild shaking at 300 rpm. To stop the reaction, cell suspensions were placed on ice. Cells were then removed by centrifugation (1000× *g* for 5 min at 4 °C). Cell supernatants were clarified of proteins using a centrifugal filter device with a 10 kDa cut-off (Vivaspin). Clarified supernatants were split into twin samples (50 µL of the original cell supernatant containing 25 nmol of NAD^+^ or cADRP) and analyzed by reversed-phase HPLC, with and without an ion-pair reagent on the 1200 and 1260 Series systems from Agilent Technologies. cADPR was analyzed by the reversed-phase HPLC on a C-8 Luna column (Phenomenex) with buffer A (20mM KHPO_4_, pH 6), a flow rate of 0.8 mL/min, and the following gradient: 0–5 min, 0% MeOH; 27.5–30 min, 50% MeOH; 32–43 min, 0% MeOH. All other metabolites were analyzed by reversed-phase HPLC with the ion-pair reagent tetrabutylammonium dihydrogen phosphate (TBAHP) on a Multohyp BDS C18 column (250 mm, 4.6 mm, particle size 5 μm; Chromatographie Service) with buffer A (20 mM KHPO_4_, 5 mM TBAHP, pH 6), and the following gradient: 0 min, 15% MeOH; 3.5 min, 15% MeOH; 11–15 min, 31.25% MeOH; 25–27 min, 50% MeOH; 29–38 min, and 15% MeOH. In both cases, the flow rate was 0.8 mL/min. Absorbance was measured at 260 nm using the DAD detector of the Agilent systems and data were processed using the ChemStation (Rev. C.01.05; Agilent Technologies, Santa Clara, CA, USA). Peaks were identified by comparing their retention time to standards. For NAD^+^, ADPR, and cADPR, the standard curves were constructed by plotting the “area under the curve” against the amount of substance for three to five standards with different concentrations. Using the slope from these standard curves, the amount of nucleotides in the samples was determined.

## 5. Conclusions

Daratumumab and hcAb MU1067 inhibit the ADPR cyclase but not the NAD^+^ hydrolase activity of the CD38-expressing MM cells. We conclude that neither clinically approved daratumumab nor recently developed nanobody-derived hcAbs provide a second mode of action against MM cells. Thus, there remains a quest for “double action” CD38-inhibitory antibodies.

## 6. Patents

The CD38-specific nanobodies reported here are covered by the patent WO2017081211A2.

## Data Availability

Data is contained within the article.

## References

[1] Lomas O.C., Tahri S., Ghobrial I.M. (2020). The microenvironment in myeloma. Curr. Opin. Oncol..

[2] Holthof L.C., Mutis T. (2020). Challenges for Immunotherapy in Multiple Myeloma: Bone Marrow Microenvironment-Mediated Immune Suppression and Immune Resistance. Cancers.

[3] Horenstein A.L., Bracci C., Morandi F., Malavasi F. (2019). CD38 in Adenosinergic Pathways and Metabolic Re-programming in Human Multiple Myeloma Cells: In-tandem Insights from Basic Science to Therapy. Front. Immunol..

[4] Paiva B., Pérez-Andrés M., Vídriales M.B., Almeida J., de las Heras N., Mateos M.V., López-Corral L., Gutiérrez N.C., Blanco J., Oriol A. (2011). Competition between clonal plasma cells and normal cells for potentially overlapping bone marrow niches is associated with a progressively altered cellular distribution in MGUS vs myeloma. Leukemia.

[5] Yang R., Elsaadi S., Misund K., Abdollahi P., Vandsemb E.N., Moen S.H., Kusnierczyk A., Slupphaug G., Standal T., Waage A. (2020). Conversion of ATP to adenosine by CD39 and CD73 in multiple myeloma can be successfully targeted together with adenosine receptor A2A blockade. J. Immunother. Cancer.

[6] Horenstein A.L., Chillemi A., Zaccarello G., Bruzzone S., Quarona V., Zito A., Serra S., Malavasi F. (2013). A CD38/CD203a/CD73 ectoenzymatic pathway independent of CD39 drives a novel adenosinergic loop in human T lymphocytes. Oncoimmunology.

[7] Konen J.M., Fradette J.J., Gibbons D.L. (2019). The Good, the Bad and the Unknown of CD38 in the Metabolic Microenvironment and Immune Cell Functionality of Solid Tumors. Cells.

[8] Zocchi E., Franco L., Guida L., Benatti U., Bargellesi A., Malavasi F., Lee H.C., De Flora A. (1993). A single protein immunologically identified as CD38 displays NAD^+^ glycohydrolase, ADP-ribosyl cyclase and cyclic ADP-ribose hydrolase activities at the outer surface of human erythrocytes. Biochem. Biophys. Res. Commun..

[9] Howard M., Grimaldi J.C., Bazan J.F., Lund F.E., Santos-Argumedo L., Parkhouse R.M., Walseth T.F., Lee H.C. (1993). Formation and hydrolysis of cyclic ADP-ribose catalyzed by lymphocyte antigen CD38. Science.

[10] Schuber F., Lund F.E. (2004). Structure and enzymology of ADP-ribosyl cyclases: Conserved enzymes that produce multiple calcium mobilizing metabolites. Curr. Mol. Med..

[11] Munshi C., Aarhus R., Graeff R., Walseth T.F., Levitt D., Lee H.C. (2000). Identification of the enzymatic active site of CD38 by site-directed mutagenesis. J. Biol. Chem..

[12] Lee H.C. (2006). Structure and enzymatic functions of human CD38. Mol. Med..

[13] Prasad G.S., McRee D.E., Stura E.A., Levitt D.G., Lee H.C., Stout C.D. (1996). Crystal structure of Aplysia ADP ribosyl cyclase, a homologue of the bifunctional ectozyme CD38. Nat. Struct. Biol..

[14] Liu Q., Kriksunov I.A., Graeff R., Munshi C., Lee H.C., Hao Q. (2005). Crystal structure of human CD38 extracellular domain. Structure.

[15] Liu Q., Graeff R., Kriksunov I.A., Jiang H., Zhang B., Oppenheimer N., Lin H., Potter B.V., Lee H.C., Hao Q. (2009). Structural basis for enzymatic evolution from a dedicated ADP-ribosyl cyclase to a multifunctional NAD hydrolase. J. Biol. Chem..

[16] Graeff R., Liu Q., Kriksunov I.A., Kotaka M., Oppenheimer N., Hao Q., Lee H.C. (2009). Mechanism of cyclizing NAD to cyclic ADP-ribose by ADP-ribosyl cyclase and CD38. J. Biol. Chem..

[17] Chini E.N., Chini C.C.S., Espindola Netto J.M., de Oliveira G.C., van Schooten W. (2018). The Pharmacology of CD38/NADase: An Emerging Target in Cancer and Diseases of Aging. Trends Pharm. Sci..

[18] Jiao Y., Yi M., Xu L., Chu Q., Yan Y., Luo S., Wu K. (2020). CD38: Targeted therapy in multiple myeloma and therapeutic potential for solid cancers. Expert Opin. Investig. Drugs.

[19] Calabretta E., Carlo-Stella C. (2020). The Many Facets of CD38 in Lymphoma: From Tumor-Microenvironment Cell Interactions to Acquired Resistance to Immunotherapy. Cells.

[20] Bannas P., Hambach J., Koch-Nolte F. (2017). Nanobodies and Nanobody-Based Human Heavy Chain Antibodies as Antitumor Therapeutics. Front. Immunol..

[21] de Weers M., Tai Y.T., van der Veer M.S., Bakker J.M., Vink T., Jacobs D.C., Oomen L.A., Peipp M., Valerius T., Slootstra J.W. (2011). Daratumumab, a novel therapeutic human CD38 monoclonal antibody, induces killing of multiple myeloma and other hematological tumors. J. Immunol..

[22] Lokhorst H.M., Plesner T., Laubach J.P., Nahi H., Gimsing P., Hansson M., Minnema M.C., Lassen U., Krejcik J., Palumbo A. (2015). Targeting CD38 with Daratumumab Monotherapy in Multiple Myeloma. N. Engl. J. Med..

[23] Lonial S., Weiss B.M., Usmani S.Z., Singhal S., Chari A., Bahlis N.J., Belch A., Krishnan A., Vescio R.A., Mateos M.V. (2016). Daratumumab monotherapy in patients with treatment-refractory multiple myeloma (SIRIUS): An open-label, randomised, phase 2 trial. Lancet.

[24] Sidaway P. (2018). Haematological cancer: Daratumumab proves effective in patients with newly diagnosed multiple myeloma. Nat. Rev. Clin. Oncol..

[25] Bhatnagar V., Gormley N.J., Luo L., Shen Y.L., Sridhara R., Subramaniam S., Shen G., Ma L., Shord S., Goldberg K.B. (2017). FDA Approval Summary: Daratumumab for Treatment of Multiple Myeloma After One Prior Therapy. Oncologist.

[26] Dimopoulos M.A., Oriol A., Nahi H., San-Miguel J., Bahlis N.J., Usmani S.Z., Rabin N., Orlowski R.Z., Komarnicki M., Suzuki K. (2016). Daratumumab, Lenalidomide, and Dexamethasone for Multiple Myeloma. N. Engl. J. Med..

[27] Palumbo A., Chanan-Khan A., Weisel K., Nooka A.K., Masszi T., Beksac M., Spicka I., Hungria V., Munder M., Mateos M.V. (2016). Daratumumab, Bortezomib, and Dexamethasone for Multiple Myeloma. N. Engl. J. Med..

[28] Fedyk E.R., Zhao L., Koch A., Smithson G., Estevam J., Chen G., Lahu G., Roepcke S., Lin J., McLean L. (2020). Safety, tolerability, pharmacokinetics and pharmacodynamics of the anti-CD38 cytolytic antibody TAK-079 in healthy subjects. Br. J. Clin. Pharm..

[29] Mikhael J., Richter J., Vij R., Cole C., Zonder J., Kaufman J.L., Bensinger W., Dimopoulos M., Lendvai N., Hari P. (2020). A dose-finding Phase 2 study of single agent isatuximab (anti-CD38 mAb) in relapsed/refractory multiple myeloma. Leukemia.

[30] Raab M.S., Engelhardt M., Blank A., Goldschmidt H., Agis H., Blau I.W., Einsele H., Ferstl B., Schub N., Röllig C. (2020). MOR202, a novel anti-CD38 monoclonal antibody, in patients with relapsed or refractory multiple myeloma: A first-in-human, multicentre, phase 1-2a trial. Lancet Haematol..

[31] Roepcke S., Plock N., Yuan J., Fedyk E.R., Lahu G., Zhao L., Smithson G. (2018). Pharmacokinetics and pharmacodynamics of the cytolytic anti-CD38 human monoclonal antibody TAK-079 in monkey-model assisted preparation for the first in human trial. Pharm. Res. Perspect..

[32] Li T., Qi S., Unger M., Hou Y.N., Deng Q.W., Liu J., Lam C.M.C., Wang X.W., Xin D., Zhang P. (2016). Immuno-targeting the multifunctional CD38 using nanobody. Sci. Rep..

[33] Schütze K., Petry K., Hambach J., Schuster N., Fumey W., Schriewer L., Röckendorf J., Menzel S., Albrecht B., Haag F. (2018). CD38-Specific Biparatopic Heavy Chain Antibodies Display Potent Complement-Dependent Cytotoxicity Against Multiple Myeloma Cells. Front. Immunol..

[34] Hambach J., Riecken K., Cichutek S., Schütze K., Albrecht B., Petry K., Röckendorf J.L., Baum N., Kröger N., Hansen T. (2020). Targeting CD38-Expressing Multiple Myeloma and Burkitt Lymphoma Cells In Vitro with Nanobody-Based Chimeric Antigen Receptors (Nb-CARs). Cells.

[35] Schriewer L., Schütze K., Petry K., Hambach J., Fumey W., Koenigsdorf J., Baum N., Menzel S., Rissiek B., Riecken K. (2020). Nanobody-based CD38-specific heavy chain antibodies induce killing of multiple myeloma and other hematological malignancies. Theranostics.

[36] An N., Hou Y.N., Zhang Q.X., Li T., Zhang Q.L., Fang C., Chen H., Lee H.C., Zhao Y.J., Du X. (2018). Anti-Multiple Myeloma Activity of Nanobody-Based Anti-CD38 Chimeric Antigen Receptor T Cells. Mol. Pharm..

[37] Deckert J., Wetzel M.C., Bartle L.M., Skaletskaya A., Goldmacher V.S., Vallée F., Zhou-Liu Q., Ferrari P., Pouzieux S., Lahoute C. (2014). SAR650984, a novel humanized CD38-targeting antibody, demonstrates potent antitumor activity in models of multiple myeloma and other CD38+ hematologic malignancies. Clin. Cancer Res. Off. J. Am. Assoc. Cancer Res..

[38] Moreno L., Perez C., Zabaleta A., Manrique I., Alignani D., Ajona D., Blanco L., Lasa M., Maiso P., Rodriguez I. (2019). The Mechanism of Action of the Anti-CD38 Monoclonal Antibody Isatuximab in Multiple Myeloma. Clin. Cancer Res. Off. J. Am. Assoc. Cancer Res..

[39] Overdijk M.B., Verploegen S., Bögels M., van Egmond M., Lammerts van Bueren J.J., Mutis T., Groen R.W., Breij E., Martens A.C., Bleeker W.K. (2015). Antibody-mediated phagocytosis contributes to the anti-tumor activity of the therapeutic antibody daratumumab in lymphoma and multiple myeloma. MAbs.

[40] Zhu C., Song Z., Wang A., Srinivasan S., Yang G., Greco R., Theilhaber J., Shehu E., Wu L., Yang Z.Y. (2020). Isatuximab Acts Through Fc-Dependent, Independent, and Direct Pathways to Kill Multiple Myeloma Cells. Front. Immunol..

[41] Abramson H.N. (2018). Monoclonal Antibodies for the Treatment of Multiple Myeloma: An Update. Int. J. Mol. Sci..

[42] Hammami A., Allard D., Allard B., Stagg J. (2019). Targeting the adenosine pathway for cancer immunotherapy. Semin. Immunol..

[43] Thompson E.A., Powell J.D. (2020). Inhibition of the Adenosine Pathway to Potentiate Cancer Immunotherapy: Potential for Combinatorial Approaches. Annu. Rev. Med..

[44] de Oliveira Bravo M., Carvalho J.L., Saldanha-Araujo F. (2016). Adenosine production: A common path for mesenchymal stem-cell and regulatory T-cell-mediated immunosuppression. Purinergic Signal..

[45] Morandi F., Horenstein A.L., Chillemi A., Quarona V., Chiesa S., Imperatori A., Zanellato S., Mortara L., Gattorno M., Pistoia V. (2015). CD56brightCD16- NK Cells Produce Adenosine through a CD38-Mediated Pathway and Act as Regulatory Cells Inhibiting Autologous CD4+ T Cell Proliferation. J. Immunol..

[46] Morandi F., Morandi B., Horenstein A.L., Chillemi A., Quarona V., Zaccarello G., Carrega P., Ferlazzo G., Mingari M.C., Moretta L. (2015). A non-canonical adenosinergic pathway led by CD38 in human melanoma cells induces suppression of T cell proliferation. Oncotarget.

[47] Hogan K.A., Chini C.C.S., Chini E.N. (2019). The Multi-faceted Ecto-enzyme CD38: Roles in Immunomodulation, Cancer, Aging, and Metabolic Diseases. Front. Immunol..

[48] Horenstein A.L., Quarona V., Toscani D., Costa F., Chillemi A., Pistoia V., Giuliani N., Malavasi F. (2016). Adenosine Generated in the Bone Marrow Niche through a CD38-Mediated Pathway Correlates with Progression of Human Myeloma. Mol. Med..

[49] Martin T.G., Corzo K., Chiron M., Velde H.V., Abbadessa G., Campana F., Solanki M., Meng R., Lee H., Wiederschain D. (2019). Therapeutic Opportunities with Pharmacological Inhibition of CD38 with Isatuximab. Cells.

[50] Fumey W., Koenigsdorf J., Kunick V., Menzel S., Schütze K., Unger M., Schriewer L., Haag F., Adam G., Oberle A. (2017). Nanobodies effectively modulate the enzymatic activity of CD38 and allow specific imaging of CD38(+) tumors in mouse models in vivo. Sci. Rep..

[51] van de Donk N.W., Janmaat M.L., Mutis T., Lammerts van Bueren J.J., Ahmadi T., Sasser A.K., Lokhorst H.M., Parren P.W. (2016). Monoclonal antibodies targeting CD38 in hematological malignancies and beyond. Immunol. Rev..

[52] Graeff R.M., Walseth T.F., Hill H.K., Lee H.C. (1996). Fluorescent analogs of cyclic ADP-ribose: Synthesis, spectral characterization, and use. Biochemistry.

[53] Berthelier V., Tixier J.M., Muller-Steffner H., Schuber F., Deterre P. (1998). Human CD38 is an authentic NAD(P)+ glycohydrolase. Biochem. J..

[54] Muller-Steffner H.M., Malver O., Hosie L., Oppenheimer N.J., Schuber F. (1992). Slow-binding inhibition of NAD^+^ glycohydrolase by arabino analogues of beta-NAD. J. Biol. Chem..

[55] Chapuy C.I., Aguad M.D., Nicholson R.T., AuBuchon J.P., Cohn C.S., Delaney M., Fung M.K., Unger M., Doshi P., Murphy M.F. (2016). International validation of a dithiothreitol (DTT)-based method to resolve the daratumumab interference with blood compatibility testing. Transfusion.

[56] De Genst E., Silence K., Decanniere K., Conrath K., Loris R., Kinne J., Muyldermans S., Wyns L. (2006). Molecular basis for the preferential cleft recognition by dromedary heavy-chain antibodies. Proc. Natl. Acad. Sci. USA.

[57] Wesolowski J., Alzogaray V., Reyelt J., Unger M., Juarez K., Urrutia M., Cauerhff A., Danquah W., Rissiek B., Scheuplein F. (2009). Single domain antibodies: Promising experimental and therapeutic tools in infection and immunity. Med. Microbiol. Immunol..

[58] Desmyter A., Transue T.R., Ghahroudi M.A., Thi M.H., Poortmans F., Hamers R., Muyldermans S., Wyns L. (1996). Crystal structure of a camel single-domain VH antibody fragment in complex with lysozyme. Nat. Struct. Biol..

[59] Unger M., Eichhoff A.M., Schumacher L., Strysio M., Menzel S., Schwan C., Alzogaray V., Zylberman V., Seman M., Brandner J. (2015). Selection of nanobodies that block the enzymatic and cytotoxic activities of the binary Clostridium difficile toxin CDT. Sci. Rep..

